# Pharmacological and non-pharmacological treatments for insomnia: A protocol for a systematic review and network meta-analysis

**DOI:** 10.1097/MD.0000000000026678

**Published:** 2021-08-06

**Authors:** Wang Chun, Deng Chao, Han Qi, Zhu Dongliang, Li Zhenmei, Li Jia

**Affiliations:** a Xianning Central Hospital, The First Affiliated Hospital of Hubei University of Science and Technology, Xianning, Hubei; b Hubei University of Chinese Medicine; c College of Acupuncture and Orthopedics, Hubei University of Chinese Medicine, Wuhan, China.

**Keywords:** insomnia, meta-analysis, network meta-analysis, systematic review

## Abstract

**Background:**

Although nonpharmacological therapies are recommended as first-line treatments for insomnia, they do not widely implement in practice owing to costly or time-consuming. As a result, pharmacotherapy remains to be commonly prescribed for patients with the sleep disorder. Pharmacotherapy for insomnia consists of different types of drugs. Few studies focused on comprehensively evaluating all available drugs for insomnia. Our review aims to compare efficacy and safety of pharmacological and nonpharmacological treatments by synthesizing direct evidence and indirect evidence to help clinicians and patients make informed decisions for insomnia.

**Methods:**

We will search the MEDLINE, EMBASE, and Cochrane Register of Controlled Trials between January 2000 and June 12, 2021. Randomized controlled trials of pharmacological and nonpharmacological interventions for insomnia will be included. Study quality will be assessed on the basis of the methodology and categories described in the Cochrane Collaboration Handbook. Eight network meta-analyses were conducted. A Bayesian network meta-analysis would be performed, and relative ranking of agents would be assessed. A node splitting method will be used to examine the inconsistency between direct and indirect comparisons when a loop connecting 3 arms exists.

**Results:**

The results of this paper will be submitted to a peer-reviewed journal for publication.

**Conclusion:**

The conclusion of our study will provide updated evidence to rank the effectiveness and safety of pharmacological and nonpharmacological interventions for insomnia.

**Ethics and dissemination:**

Ethical approval is not applicable, as this study is a network meta-analysis based on published trials.

**INPLASY registration number:**

INPLASY202160031

## 1 Introduction

### 1.1 Description of the condition

Insomnia is a sleep disorder characterized by difficulty in sleep initiation, difficulty in sleep maintenance or early morning awakening, occurring at least 3 times per week for at least 3 months, with significant personal distress, and daytime functional impairments.^[1]^ Insomnia has become the most common sleep disorder disease in the population, and it is also the most common disease in sleep disorder clinic. Due to life pressures, rates of insomnia have increased, insomnia has been reported that the average insomnia rate in the world is 27%, as high as 31.2% in China, and the persistent rate of insomnia in adults is 30% to 60%. About 50% of patients will develop into chronic insomnia.^[2]^ These patients suffer from impaired daytime function and a series of physiological dysfunctions.

The causes of insomnia are multifactorial. Several psychophysiological and behavioral constructs considered “hyperarousal” a key component in etiology and pathophysiology of insomnia.^[3]^ Hyperarousal model of insomnia postulates that high arousal would alter individual's cognitive, physiological, and psychological states, leading to disturbed sleep.^[3,4]^ the Spielman's “3-P” model delineates the development of insomnia through the progression from acute to chronic insomnia as a result of three closely interactive factors: predisposing, precipitating, and perpetuating factors. Predisposing factors refer to the genetic, physiological, and psychological risk factors that may increase individual's vulnerability to the onset of insomnia. Precipitating factors include the physiological, psychological, and environmental stressors that may trigger the acute onset of sleep disturbance. Perpetuating factors refer to the distorted cognitions about sleep and insomnia, and maladaptive behaviors that individuals with insomnia tend to adopt as a way to cope with their sleep problem, but often inadvertently exacerbate their sleep difficulty.^[1]^

### 1.2 Description of the interventions

A number of different nonpharmacological and pharmacological treatments have been used to manage insomnia in clinical practice, which lead to great challenges for clinicians to make appropriate decisions.

#### 1.2.1 Cognitive behavioral therapy for insomnia (CBT-I) and other psychotherapeutic approaches

CBT-I has emerged as the most prominent nonpharmacological treatment approach and is recommended as the first-line treatment for chronic insomnia in adults by American Academy of Sleep Medicine,^[5,6]^ CBT-I is a multi-component treatment targeting behavioral, cognitive, and physiological factors that perpetuate insomnia and aims to modify and alter maladaptive behaviors and distorted beliefs about sleep and insomnia and can be delivered in different formats, such as face-to-face individual or group therapy, guided or unguided digitally delivered self-help format.^[7,8]^ The evaluation of CBT-I to treat insomnia has been summarized in meta-analytic studies that showed that CBT-I has a positive impact on both insomnia complaints and comorbid symptoms.^[9,10]^

#### 1.2.2 Pharmacotherapy

Multiple prescription drugs and over-the-counter (OTC) products are currently used to treat insomnia, and these include benzodiazepines (BZDs), antidepressants, antihistamines, melatonin, and phytotherapeutic substances.^[11]^ Meta-analyses of Huedo-Medina et al^[12]^ and Winkler et al^[13]^ clearly show that BZ and BZRAs are effective in the short-term treatment (≤4 weeks) of insomnia. Pillai et al^[14]^ analyzed data from 1 randomized controlled trial with BZRAs according to definitions of treatment response/remission, and observed positive treatment responses in 76.7% of cases and remissions in 47.7% of participants. There are insufficient evidences on the efficacy of antihistamines in insomnia. A systematic review concluded that antihistamines have only a small to moderate efficacy in the treatment of insomnia and that tolerance to these substances develops quickly.^[15]^ Some antidepressants such as doxepin are used in the treatment of insomnia and the dosages for antidepressants to treat insomnia are usually much lower than the recommended doses for depression. Some evidences concluded that the efficacy of sedating antidepressants is weaker than that for BZ/BZRAs.^[13,16]^ Melatonin is a hormone produced in the pineal gland under control of the circadian system in the hypothalamic suprachiasmatic nucleus (SCN).^[17]^ Ramelteon is a selective agonist for the MT1 and MT2 melatonin receptors that are highly represented in the SCN. Buscemi et al^[18]^ and Ferracioli-Oda et al^[19]^ reported that melatonin reduces sleep-onset latency, which was also demonstrated for ramelteon. Kuriyama et al^[20]^ also found significant positive effects of melatonin on sleep-onset latency and sleep quality. The efficacy of phytotherapeutics in the treatment of insomnia has been explored in several researches. A meta-analysis of studies investigating Chinese herbal medicine (CHM) concluded that CHM is superior to placebo with respect to its effect on subjective sleep parameters and equally effective as BZ.^[21]^ But at present, the methodological quality of the studies was poor and further studies are warranted.

#### 1.2.3 Light therapy and exercise

Light exposure therapy is a nonpharmacological intervention. Outside light stimulus may affect sleep mode.^[22]^ For example, light exposure during the evening can delay sleep onset, while light exposure in the early morning can anticipate sleep onset. Furthermore, the human circadian system is more sensitive to short-wavelength (blue) light, although at higher intensity, no much difference is found between blue enriched light and white broad spectrum light.^[23]^ Ocular exposition to bright light is capable of phase shifting the circadian rhythms in humans. Consequently, it has been proposed as a treatment for those disorders in which a circadian alteration is central. Exercise doubtlessly has positive effects on psychological and physical health, and many studies show that regular exercise reduces mortality.^[24]^ Of particular importance for the current guideline, both light therapy and exercise have also been suggested to be efficacious in patients with insomnia.

#### 1.2.4 Complementary and alternative interventions

In the area of complementary and alternative medicine, several treatments for insomnia have been suggested, including acupuncture, acupressure, aromatherapy, foot reflexology, homeopathy, meditative movement therapies, moxibustion, music therapy, and yoga. Some evidence proved that complementary and alternative medicine has great potential to treat patients suffered from insomnia. Chen et al^[25]^ found that auricular acupuncture has positive effect in the treatment of insomnia. Yeung et al^[26]^ found that comparing with sham acupuncture, acupuncture was more efficacious.

In general terms, the numerous alternative options exist for insomnia in clinical practice. However, the reliability of these evidence varied greatly, some of them based on outdated SRs, which just included small sample size and concluded imprecise and different results, and some others just based on RCTs without evaluation of risk of bias, all these discrepancies could lead to obstacles and challenges for physicians to give optimal management for insomnia; thus, a comprehensive comparisons is necessary to rank all available pharmacological and nonpharmacological interventions and their combinations based on effectiveness and safety outcomes. The comprehensive comparisons should include direction comparisons and indirection comparisons of available RCTs, that is, conducting a network meta-analysis (NMA).

## 2 Method and design

This protocol is registered in INPLASY (INPLASY202160031). This is an SRs and MA of clinical studies, so ethical approval is not necessary.

### 2.1 Data sources

A comprehensive search of studies published between January 2000 and June 12, 2021, was conducted in the PubMed, Embase, and Cochrane Register of Controlled Trials databases using the keywords “Primary Insomnia” OR “Insomnia, Primary,” OR “Chronic Insomnia,” OR “Insomnia, Chronic,” OR “Sleep Initiation and Maintenance Disorders,” OR “Sleep disturbances,” OR “Sleep disorder,” OR “Sleep Initiation Dysfunction.” Furthermore, the references of the included studies and related review were checked for additional relevant studies. Studies that satisfied the following conditions were included in the meta-analysis: Population: Individuals with insomnia disorder of all ages (including adult and pediatric populations) and of both gender with or without any mental, somatic or sleep comorbidity; Intervention: experimental interventions administered alone; Comparison: waiting list, no treatment, pharmacological and psychological (e.g., psychoeducation) placebo, standard therapy for insomnia: sleep pharmacotherapy [hypnotics: benzodiazepine (BZ) and benzodiazepine receptor agonists (BZRA) and recommended psychological treatment, i.e., CBT-I (CBT-I, sleep restriction, stimulus control]; Outcomes: objective and subjective standardized measures of sleep and/or insomnia. Study design: Randomized controlled trial. The study was published in a peer-reviewed English language journal. For longitudinal studies, only the baseline data were included. For studies reporting both on- and off-state results, only the off-state datasets were included. In cases wherein patient datasets overlapped between separate articles, only the dataset with the largest sample size and the most comprehensive information was included. The corresponding author of each included study was contacted via email when additional information was required.

### 2.2 Data extraction

Descriptive information related to sample demographic and clinical characteristics, clinical aspects of insomnia disorder, intervention characteristics and methodological aspects of the study design was collected. Descriptive information included age and sex of participants in the experimental and control interventions; diagnostic tool/s according to which participants met the definition of insomnia disorder; past medical conditions; information about mental and somatic comorbidities; number of treatment sessions and duration; treatment setting and conducting personnel; type of control intervention; insomnia duration.

**Figure 1:**
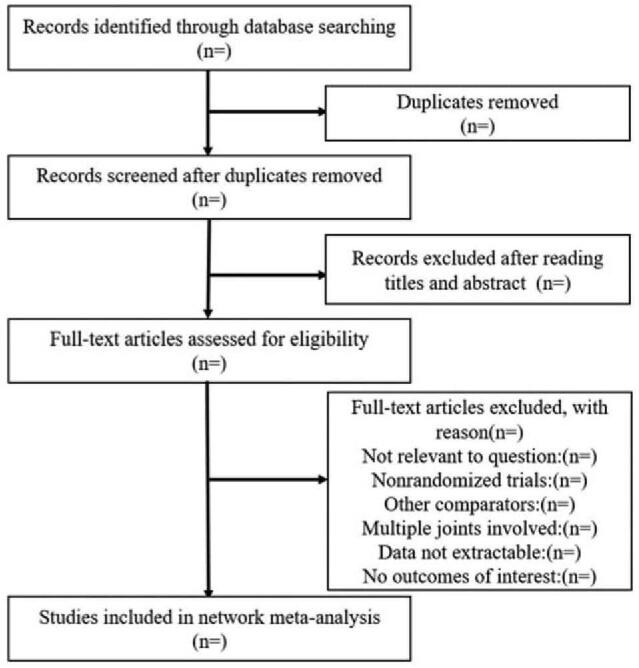
Flow diagram of including studies.

Interventions were coded independently by a clinician and a methodologist using a pre-established coding. Included interventions were classified into the following 2 categories: pharmacological interventions and nonpharmacological interventions. The results of electronic searches will be managed using Endnote X9. Two reviewers (Wang Chun and Li Zhenmei) will review articles identified from different databases according to eligibility criteria independently. Duplicates will be first removed. A third reviewer (Zhu Dongliang) made the final decision when there is a disagreement between two researchers. The entire process of selecting studies is shown in the PRISMA flow diagram (Fig. 1).

### 2.3 Assessment of risk of bias

Two reviewers will evaluate the risk of bias of the selected RCTs according to the criteria and technique proposed in the Cochrane Handbook V.5.2.0,^[27]^ which includes random sequence generation (selection bias), allocation concealment (selection bias), blinding of participants and personnel (performance bias), blinding of outcome assessment (detection bias), incomplete outcome data (attrition bias), selective reporting (reporting bias), and other bias. Each study will be assigned a level of risk of bias (high risk, unclear risk, low risk) for each item. Any disagreement will be resolved through discussion or consultation with an independent third adjudicator.

### 2.4 Assessment of the quality of evidence

Using the Grading of Recommendations Assessment, Development and Evaluation (GRADE), the quality of evidence will be evaluated as 4 levels—high quality, moderate quality, low quality, and very low quality.^[28]^ This process will be performed with the online guideline development tool (GDT, http://gdt.guidelinedevelopment.org/).

### 2.5 Statistical analyses

Analyses were planned for an application for funding by the German Ministry for Education and Research (Bundesministerium für Bildung und Forschung, BMBF, 01KG1111). The plan was structured together with a biostatistician with excellent expertise in network meta-analyses (G.R.). Data were extracted both for sleep and insomnia related variables as well as for daytime symptoms. Data are available in the online Supplementary Materials online (Document S2: Data extraction). Nevertheless, neither statistical analysis nor qualitative summary was conducted for data related to daytime symptoms, as these were too few and too different between studies. Eight network meta-analyses were conducted considering experimental and control interventions categories as explained above (Data extraction) for the following categories of outcomes.

(1) Self-reported sleep efficiency: defined as a sleep efficiency index from sleep diaries or, if this was not reported, as sleep quality perception from sleep diaries;
(2) Sleep efficiency measured through physiological indices: defined as sleep efficiency index measured by polysomnography, or, if this was not reported, by actigraphy;
(3) Self-reported daytime sleepiness: defined as total score of the Epworth Sleepiness Scale,^[29]^ or, if this was not available, of the Children's Sleep Habits Scale (daytime symptoms which includes a scale of daytime sleepiness^[30]^);
(4) Self-reported severity of sleep problems: defined as total score from Insomnia Severity Index,^[31]^ or, if this was not available, from the Pittsburgh Sleep Quality Index.^[32]^
(5) Self-reported sleep onset latency: defined as sleep onset latency measured through sleep diaries;
(6) Sleep onset latency measured through physiological indices: defined as sleep onset latency measured by polysomnography or, if this was not reported, by actigraphy.
(7) Self-reported wake time during the night: defined as wake after sleep onset latency measured through sleep diaries;
(8) Wake time during the night measured through physiological indices: defined as wake after sleep onset latency measured by polysomnography or, if this was not reported, by actigraphy.

Meta-analytic calculations were performed with the statistical software package R (http://www.Rproject.org/). A frequentist NMA was performed using the R-package “netmeta.” Through NMA, it is possible to graphically represent the comparisons included in the network. The function “netgraph” was used to visualize a net-graph evidencing the geometry of the network. The network graph consists of nodes that represent the treatments and edges that represent direct evidence, that is, treatments that are directly compared in at least one study.

Second, we calculated effect sizes as standardized mean differences (Cohens’d) between post-treatment assessment and baseline. We referred to data of participants who completed the post-treatment assessment. All classes of interventions were compared against placebo/waiting list interventions because this category of control treatment was used most frequently. A random-effects model was used because of expected considerable heterogeneity between studies (e.g., comorbidities, different treatment variables, and different populations).

To test the heterogeneity of the network, Cochran Q and Higgin *I*^2^ were calculated. Cochran Q is computed as a weighted sum of squared differences between single study effects and the pooled effect across studies. Significant values indicate a high level of heterogeneity between studies that needs to be further investigated. Higgin *I*^2^ calculates the variability in effect estimates that is due to between-study heterogeneity rather than due to chance. Low percentages of *I*^2^ are indicative of low heterogeneity, while percentages over 75% represent considerable levels of heterogeneity.^[33]^

In network meta-analyses, net heat plots can be plotted to investigate potential sources of heterogeneity.^[34]^ These are graphical tools that represent changes in heterogeneity due to relaxing the consistency assumption for single designs in a matrix visualization. In the matrix, areas of the grey squares indicate the contribution of pooled direct evidence of each single design in the column to each network estimate in the row. This tool also graphically represents changes in Cochran Q statistic due to relaxing the consistency assumption for single designs in matrix visualization. “Hot spots” of inconsistency are indicated by red colors, while blue colors indicate consistency.

## 3 Discussion

The purpose of this project is to provide reliable evidence of pharmacological and nonpharmacological treatments for insomnia. Researchers will rank the effectiveness and safety of the potential interventions for insomnia according to the characteristics of patients by conducting an advanced NMA based on Bayesian statistical model. In order to promote clinical practice, researchers will cooperate with the guideline development panel to translate the findings of NMA into recommendations in the future insomnia guidelines.

## Author contributions

All authors participated in the review of the manuscript; Wang Chun writes the protocol, Wang Chun and Zhu Dongliang conceived and designed the protocol, Li Zhenmei, Zhu Dongliang, Han Q reviewed the search strategy, Deng Chao and Li Jia registered the protocol in INPLASY, Li Jia revised the manuscript.

**Conceptualization:** Wang Chun, Li Jia.

**Data curation:** Wang Chun.

**Funding acquisition:** Li Jia.

**Investigation:** Deng Chao.

**Methodology:** Han Qi.

**Project administration:** Zhu Dongliang.

**Resources:** Zhu Dongliang.

**Software:** Li Zhenmei.

**Supervision:** Li Jia.

**Validation:** Li Jia.

**Visualization:** Li Jia.

**Writing – original draft:** Wang Chun.

**Writing – review & editing:** Li Jia.

## References

[1] Chan NY Chan JWY Li SX Wing YK. Non-pharmacological approaches for management of insomnia. Neurotherapeutics 2021;18:32–43. 33821446 10.1007/s13311-021-01029-2 PMC8116473

[2] Liu F-G Tan A-H Peng C-Q. Efficacy and safety of scalp acupuncture for insomnia: a systematic review and meta-analysis. eCAM 2021;2021:6621993. 34122601 10.1155/2021/6621993 PMC8166479

[3] Riemann D Spiegelhalder K Feige B. The hyperarousal model of insomnia: a review of the concept and its evidence. Sleep Med Rev 2010;14:19–31. 19481481 10.1016/j.smrv.2009.04.002

[4] Bonnet MH Arand DL. Hyperarousal and insomnia: state of the science. Sleep Med Rev 2010;14:09–15. 10.1016/j.smrv.2009.05.002 19640748

[5] Morgenthaler T Kramer M Alessi C. Practice parameters for the psychological and behavioral treatment of insomnia: an update. An American academy of sleep medicine report. Sleep 2006;29:1415–9. 17162987

[6] Sateia MJ Buysse DJ Krystal AD Neubauer DN Heald JL. Clinical practice guideline for the pharmacologic treatment of chronic insomnia in adults: an American Academy of Sleep Medicine Clinical Practice Guideline. J Clin Sleep Med 2017;13:307–49. 27998379 10.5664/jcsm.6470 PMC5263087

[7] Portenoy RK Itri LM. Cancer-related fatigue: guidelines for evaluation and management. Oncologist 1999;4:01–10. 10337366

[8] Morin CM Bootzin RR Buysse DJ. Psychological and behavioral treatment of insomnia: update of the recent evidence (1998–2004). Sleep 2006;29:1398–414. 17162986 10.1093/sleep/29.11.1398

[9] Morin CM Culbert JP Schwartz SM. Nonpharmacological interventions for insomnia: a meta-analysis of treatment efficacy. Am J Psychiatry 1994;151:1172–80. 8037252 10.1176/ajp.151.8.1172

[10] Murtagh DR Greenwood KM. Identifying effective psychological treatments for insomnia: a meta-analysis. J Consult Clin Psychol 1995;63:79–89. 7896994 10.1037//0022-006x.63.1.79

[11] Riemann D Baglioni C Bassetti C. European guideline for the diagnosis and treatment of insomnia. J Sleep Res 2017;26:675–700. 28875581 10.1111/jsr.12594

[12] Huedo-Medina TB Kirsch I Middlemass J. Effectiveness of non-benzodiazepine hypnotics in treatment of adult insomnia: meta-analysis of data submitted to the Food and Drug Administration. BMJ 2012;345:e8343. 23248080 10.1136/bmj.e8343 PMC3544552

[13] Winkler A Auer C Doering BK Rief W. Drug treatment of primary insomnia: a meta-analysis of polysomnographic randomized controlled trials. CNS Drugs 2014;28:799–816. 25168785 10.1007/s40263-014-0198-7

[14] Pillai V Roth T Roehrs T. Effectiveness of benzodiazepine receptor agonists in the treatment of insomnia: an examination of response and remission rates. Sleep 2017;40: 10.1093/sleep/zsw044 PMC6251695 28364510

[15] Vande Griend JP Anderson SL. Histamine-1 receptor antagonism for treatment of insomnia. J Am Pharm Assoc (2003) 2012;52:e210–9. 23229983 10.1331/JAPhA.2012.12051

[16] Buscemi N Vandermeer B Friesen C. The efficacy and safety of drug treatments for chronic insomnia in adults: a meta-analysis of RCTs. J Gen Intern Med 2007;22:1335–50. 17619935 10.1007/s11606-007-0251-z PMC2219774

[17] Zee PC Manthena P. The brain's master circadian clock: implications and opportunities for therapy of sleep disorders. Sleep Med Rev 2007;11:59–70. 16973392 10.1016/j.smrv.2006.06.001

[18] Buscemi N Vandermeer B Hooton N. The efficacy and safety of exogenous melatonin for primary sleep disorders. A meta-analysis. J Gen Intern Med 2005;20:1151–8. 16423108 10.1111/j.1525-1497.2005.0243.x PMC1490287

[19] Ferracioli-Oda E Qawasmi A Bloch MH. Meta-analysis: melatonin for the treatment of primary sleep disorders. PLoS One 2013;8:e63773. 23691095 10.1371/journal.pone.0063773 PMC3656905

[20] Kuriyama A Honda M Hayashino Y. Ramelteon for the treatment of insomnia in adults: a systematic review and meta-analysis. Sleep Med 2014;15:385–92. 24656909 10.1016/j.sleep.2013.11.788

[21] Ni X Shergis JL Guo X. Updated clinical evidence of Chinese herbal medicine for insomnia: a systematic review and meta-analysis of randomized controlled trials. Sleep Med 2015;16:1462–81. 26611943 10.1016/j.sleep.2015.08.012

[22] Baglioni C Bostanova Z Bacaro V. A systematic review and network meta-analysis of randomized controlled trials evaluating the evidence base of melatonin, light exposure, exercise, and complementary and alternative medicine for patients with insomnia disorder. J Clin Med 2020;9: 10.3390/jcm9061949 PMC7356922 32580450

[23] Smith MR Eastman CI. Phase delaying the human circadian clock with blue-enriched polychromatic light. Chronobiol Int 2009;26:709–25. 19444751 10.1080/07420520902927742 PMC2828683

[24] Hupin D Roche F Gremeaux V. Even a low-dose of moderate-to-vigorous physical activity reduces mortality by 22% in adults aged ≥60 years: a systematic review and meta-analysis. Br J Sports Med 2015;49:1262–7. 26238869 10.1136/bjsports-2014-094306

[25] Chen HY Shi Y Ng CS. Auricular acupuncture treatment for insomnia: a systematic review. J Altern Complement Med 2007;13:669–76. 17718650 10.1089/acm.2006.6400

[26] Yeung WF Chung KF Poon MM. Acupressure, reflexology, and auricular acupressure for insomnia: a systematic review of randomized controlled trials. Sleep Med 2012;13:971–84. 22841034 10.1016/j.sleep.2012.06.003

[27] Puhan MA Schünemann HJ Murad MH. A GRADE Working Group approach for rating the quality of treatment effect estimates from network meta-analysis. BMJ 2014;349:g5630. 25252733 10.1136/bmj.g5630

[28] Begg CB Mazumdar M. Operating characteristics of a rank correlation test for publication bias. Biometrics 1994;50:1088–101. 7786990

[29] Johns MW. A new method for measuring daytime sleepiness: the Epworth sleepiness scale. Sleep 1991;14:540–5. 1798888 10.1093/sleep/14.6.540

[30] Owens JA Spirito A McGuinn M. The Children's Sleep Habits Questionnaire (CSHQ): psychometric properties of a survey instrument for school-aged children. Sleep 2000;23:1043–51. 11145319

[31] Bastien CH Vallières A Morin CM. Validation of the Insomnia Severity Index as an outcome measure for insomnia research. Sleep Med 2001;2:297–307. 11438246 10.1016/s1389-9457(00)00065-4

[32] Buysse DJ Reynolds CF 3rd Monk TH. The Pittsburgh Sleep Quality Index: a new instrument for psychiatric practice and research. Psychiatry Res 1989;28:193–213. 2748771 10.1016/0165-1781(89)90047-4

[33] Higgins JP Thompson SG. Quantifying heterogeneity in a meta-analysis. Stat Med 2002;21:1539–58. 12111919 10.1002/sim.1186

[34] König J Krahn U Binder H. Visualizing the flow of evidence in network meta-analysis and characterizing mixed treatment comparisons. Stat Med 2013;32:5414–29. 24123165 10.1002/sim.6001

